# Association between map-like redness and the risk of gastric cancer and high-risk precancerous lesions: a systematic review and meta-analysis

**DOI:** 10.3389/fonc.2026.1760001

**Published:** 2026-04-22

**Authors:** Baixiang He, Mingyue Liu, Xiaochi Ma, Sheng Huang, Xiangyun Zou, Liju Zhang, Xiyan Zhang, Tong Li, Jinglin Zhang, Zhihong Li

**Affiliations:** 1 Gastroenterology Department, Dongzhimen Hospital, Beijing University of Chinese Medicine, Beijing, China; 2 Suzhou Hospital, Xiyuan Hospital of China Academy of Chinese Medical Sciences,, Suzhou, China; 3 Dermatology Department, Xiyuan Hospital, Beijing, China; 4 Gastroenterology Department, The First Affiliated Hospital, Henan University of Chinese Medicine, Zhengzhou, China

**Keywords:** endoscopic marker, gastric cancer, map-like redness, meta-analysis, post-*Helicobacter pylori* eradication

## Abstract

**Background and aims:**

The risk of gastric cancer (GC) following *Helicobacter pylori* (HP) eradication has gained increasing attention. Map-like redness (MLR) is an easily recognizable and highly specific endoscopic feature observed after eradication. We aimed to quantify the association between MLR and the risk of GC and high-risk precancerous lesions.

**Methods:**

We conducted a systematic search in PubMed, Embase, the Cochrane Library, CNKI, Wanfang, CBM, and VIP databases up to April 23, 2025. The primary outcome was GC, and secondary outcomes were high-risk precancerous lesions defined as the Operative Link on Gastritis Assessment (OLGA) stage > II, Gastric Intestinal Metaplasia Assessment (OLGIM) stage > II, and Kimura–Takemoto (K-T) classification > C2.

**Results:**

14 studies were included (10 for GC, 2 for OLGA, 3 for OLGIM, and 3 for K–T classification). A significant association was observed between MLR and GC (ORs 4.38; 95% CI 3.45–5.57; I^2^ = 0%). MLR was also strongly associated with high-risk precancerous lesions: OLGA stage > II (ORs 10.45; 95% CI 5.98–18.25; I^2^ = 9.6%), OLGIM stage > II (ORs 10.78; 95% CI 2.02–57.60; I^2^ = 94.0%), and K-T classification > C2 (ORs 4.65; 95% CI 2.62–8.25; I^2^ = 19.3%). Subgroup analyses by region, study design, time direction, and *H. pylori* eradication status yielded consistent results (ORs range: 3.77-5.55), and leave-one-out sensitivity analyses showed stable estimates (ORs range: 4.15-4.69), confirming the robustness of the findings.

**Conclusion:**

MLR is highly associated with an increased risk of GC and high-risk precancerous lesions.

**Systematic Review Registration:**

https://www.crd.york.ac.uk/prospero/display_record.php?ID=CRD420251048039, identifier CRD420251048039.

## 1 Introduction

Gastric cancer (GC) is the fifth most common cancer and the third leading cause of cancer-related death worldwide, with over 40% of global new cases and deaths occurring in China (1). *Helicobacter pylori* (HP), classified by the World Health Organization as a Group I carcinogen, is regarded as one of the most important risk factors for GC, driving the Correa cascade from chronic gastritis, atrophy, intestinal metaplasia, and dysplasia to gastric adenocarcinoma (2). Although HP eradication significantly reduces the incidence and mortality of GC, growing evidence indicates that eradication does not completely eliminate cancer risk (3). A recent prospective study estimated the annual incidence of GC after HP eradication to be approximately 410 cases per 100,000 person-years (4). Therefore, identifying intuitive endoscopic markers to stratify patients who remain at risk of GC after eradication therapy holds important clinical significance.

Map-like redness (MLR), also known as mottled patchy erythema, is a characteristic endoscopic finding primarily observed after HP eradication, first described by Nagata et al. and later incorporated into the 2015 Kyoto Classification of Gastritis (5, 6). MLR commonly occurs along the lesser curvature of the stomach, appearing as well-demarcated areas of abnormal mucosal coloration, often with shallow depressions, and remains readily identifiable even under standard white-light imaging (WLI) (7). MLR has attracted attention as a potential endoscopic marker for increased GC susceptibility. Histologically, it reflects extensive and severe intestinal metaplasia typically formed prior to HP eradication, indicating a potentially high-risk mucosal background (5). Clinically, several studies have demonstrated significant associations between MLR and both high-risk precancerous lesions and GC (8–11). In addition, MLR has been observed to overlap with the sites of newly developed GC, further supporting its potential role as an endoscopic indicator for cancer risk assessment (12, 13).

In the present study, we performed a systematic review and meta-analysis to assess the association between MLR and the risk of GC and high-risk precancerous lesions, with subgroup analyses according to study region, design, and HP eradication status.

## 2 Methods

### 2.1 Protocol

This epidemiological systematic review and meta-analysis adhered to the PRISMA 2020 statement and followed the MOOSE guidelines (**Supplementary Table 1**) for reporting meta-analyses of observational studies. The analysis was prospectively registered on PROSPERO (registration number CRD420251048039).

### 2.2 Search strategy and study selection

We searched CNKI, Wanfang, VIP, CBM, PubMed, Cochrane Library, and Embase from inception to 23 April 2025, The search query for PubMed was: (“map-like redness”[ti/ab] OR “patchy redness”[ti/ab] OR “Kyoto classification of gastritis”[ti/ab] OR “Kyoto classification”[ti/ab]) AND (“Stomach Neoplasms”[mesh] OR “Neoplasm, Stomach”[ti/ab] OR “Stomach Neoplasm”[ti/ab] OR “Gastric Neoplasm”[ti/ab] OR “Neoplasm, Gastric”[ti/ab] OR “Cancer of Stomach”[ti/ab] OR “Cancer of the Stomach”[ti/ab] OR “Gastric Cancer”[ti/ab] OR “Cancer, Gastric”[ti/ab] OR “Stomach Cancer”[ti/ab] OR “Cancer, Stomach”[ti/ab] OR “early gastric cancer”[ti/ab] OR “gastric adenocarcinoma”[ti/ab] OR “adenocarcinoma of the stomach”[ti/ab] OR “operative link on gastritis assessment”[ti/ab] OR “operative link on gastritis intestinal metaplasia assessment”[ti/ab] OR EGC [ti/ab] OR OLGA [ti/ab] OR OLGIM [ti/ab]). This query was adapted for the other database (**Supplementary Table 2**).

We enhanced the search by reviewing the reference lists of included full-text publications, relevant reviews, and clinical practice guidelines to identify further eligible studies. No restrictions were placed on date or language status. Papers in a language other than English and Chinese were translated with the help of the Cochrane global community group. Duplicates were removed using the “Find Duplicates” function embedded in EndNote software (version 20, Clarivate Analytics, Philadelphia, United States) for Windows. Two trained researchers (XCM, MYL) independently searched the literature, screened titles and abstracts, and then checked the full texts for eligibility. Disagreements were resolved through discussion or consultation with a third researcher (BXH).

### 2.3 Eligibility criteria

Studies were included if they met the following PICOS criteria: P (Participants): Adults (≥18 years) who underwent upper gastrointestinal endoscopy. I (Intervention/Exposure): Endoscopic detection of MLR, which were defined as an erythematous lesion with a shallow depression and boundaries distinct from the background mucosa (10). C (Comparator): Participants without MLR under endoscopy. O (Outcomes): The primary outcome was GC at any stage detected by endoscopy and confirmed by histology. The secondary outcome was high-risk gastric precancerous lesions, defined as Operative Link on Gastritis Assessment (OLGA) stage > II or Operative Link on Gastric Intestinal Metaplasia Assessment (OLGIM) stage > II, and Kimura–Takemoto (K-T) classification > C2 according to previous studies (14–16). S (Study design): Cross-sectional, case-control, or cohort studies. Studies in animal experiments, reviews, editorials, case reports, and conference abstracts without full text were excluded.

### 2.4 Data extraction and quality assessment

Data extraction was performed using a pre-determined standardized form by two independent researchers (XCM, MYL), and in the case of discrepancies, an arbitrator (BXH) resolved conflicts. We recorded the following data: first author, year and country of publication, study design, time direction, sample size, sex distribution, age range, HP eradication history, endoscopic modality, and outcomes. Before formal screening and extraction, 10% of eligible studies were randomly selected for a pilot test, followed by focused discussions to resolve extraction discrepancies, thereby minimizing bias due to individual errors. If necessary, additional information was obtained by contacting the study authors.

Methodological quality was also independently assessed by two reviewers (SH and XYZ), with any disagreements resolved by an arbitrator (BXH). The Newcastle–Ottawa Scale (NOS) was used for cohort and case–control studies, while the Joanna Briggs Institute (JBI) critical appraisal checklist was applied to cross-sectional studies. NOS assesses three domains: selection, comparability, and outcome (or exposure for case–control studies), with scores ranging from 0 to 9. Studies scoring ≥7 were classified as low risk of bias (17). JBI checklist includes eight items rated as “yes”, “no”, “unclear or not applicable”, with only “yes” responses assigned 1 point. The total score ranges from 0 to 8, and studies scoring ≥5 were similarly considered at low risk of bias (18).

### 2.5 Statistical analysis

All statistical tests were two-sided, and all analyses were performed using R software (version 4.4.2, R Foundation for Statistical Computing, Vienna, Austria), with the meta package. A significance level of p < 0.05 was considered statistically significant. The Mantel–Haenszel (M-H) method was used to calculate pooled odds ratios (ORs) with 95% confidence intervals (CIs). Heterogeneity across studies was assessed using the I^2^ statistic. When I^2^ ≥ 50%, a random-effects model was used; otherwise, a fixed-effect model was applied. Regardless of heterogeneity, the predefined subgroup analyses include: geographical region, HP eradication status, study design, and time direction to explore potential sources of heterogeneity. Sensitivity analyses were conducted using the leave-one-out method. For outcomes with 10 or more studies, publication bias was evaluated by funnel plots and Egger’s regression test.

## 3 Results

### 3.1 Study selection

**Figure 1:**
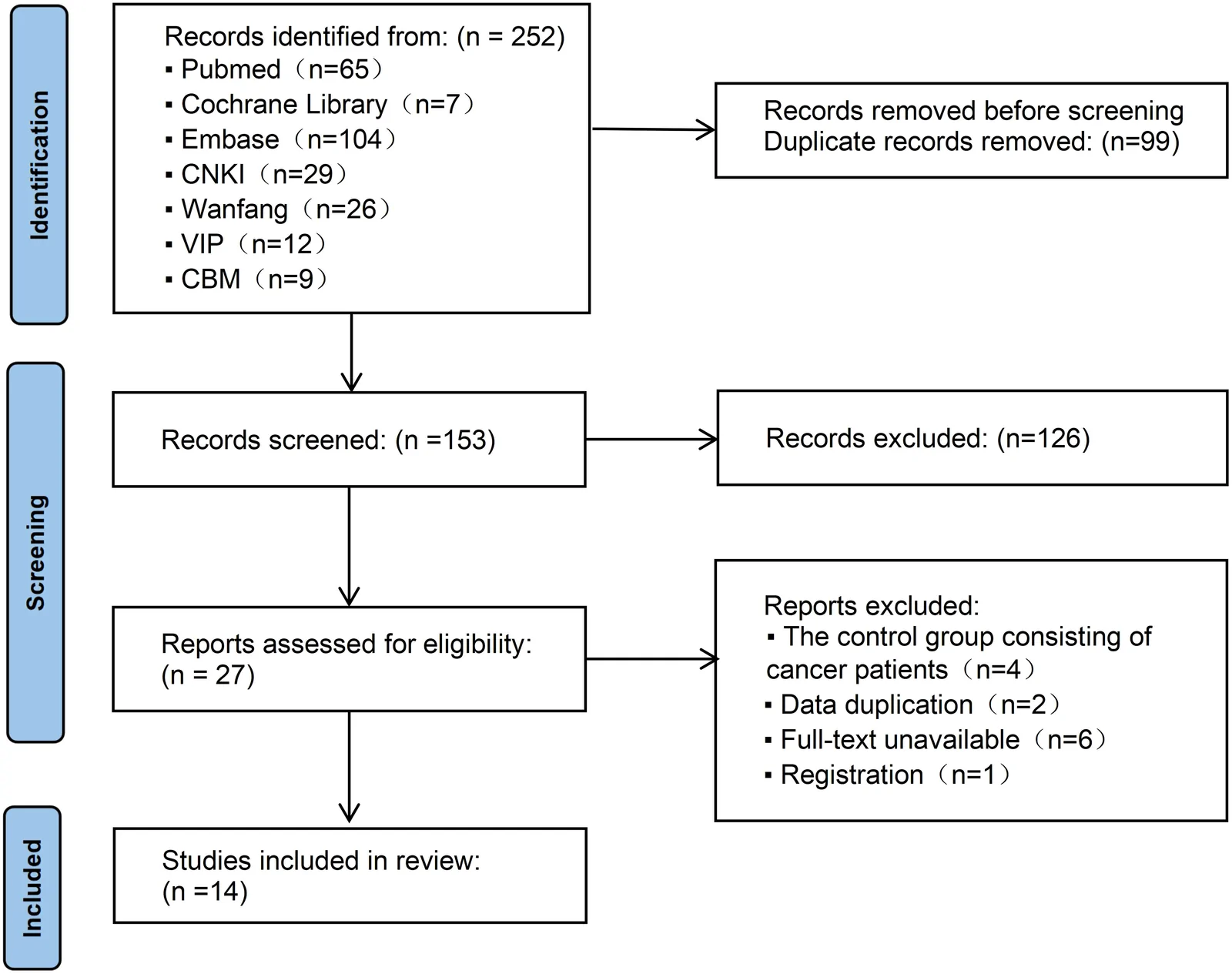
PRISMA 2020 flow diagram of the study selection process.

According to the aforementioned search strategy and after removal of duplicates, a total of 153 studies were identified. Based on the title and abstract, we selected 27 articles for full-text examination and finally included 14 studies in this meta-analysis (**Figure 1**). No additional studies were identified through manual reference screening. Of these, 10 studies (7, 10–12, 19–24) investigated the association between MLR and GC, 2 studies (8, 9) focused on OLGA staging, 3 studies (8, 9, 25) on OLGIM staging, and 3 studies (10, 24, 26) on K-T classification.

### 3.2 Characteristics of the included studies

The included 14 studies were published between 2016 and 2024, encompassing 3252 participants, with individual study sample sizes ranging from 93 to 422. Among them, 8 studies were conducted in China (7–9, 19–21, 25, 26) and 6 in Japan (10–12, 22–24), reflecting a geographic focus in East Asia. Most studies adopted a cross-sectional (10 studies) (8–11, 20, 22–26) or case-control design (3 studies) (7, 19, 21), with only one cohort study reporting an average follow-up time of 3.9 years (12). Regarding time direction, 10 studies were retrospective (7–12, 19, 22, 25, 26) and 4 were prospective (20–24). The history of HP eradication was complete in 8 studies (7, 10–12, 21, 22, 24, 26), partial in 5 studies (18.3%–80.1%) (8, 9, 19, 20, 23), and unclear in 1 study (25). MLR was identified using WLI in all studies, with several additionally incorporating image-enhanced techniques such as narrow-band imaging (9, 10, 21, 24), linked color imaging (7, 10, 11), or blue laser imaging (24). Detailed characteristics of the included studies are summarized in **Table 1**.

**Table 1:** Baseline characteristics of the included studies.

First author, year of publication	Country	Study design	Endoscopy	Sample size	Female, %	Age, years mean age (SD)	HP eradication (%)	Outcome
Gao, 2024 (19)	China	Case-control, R	WLI	360	36.1	–	18.3	GC
Zhang, 2023a (7)	China	Case-control, R	WLI+LCI	138	23.2	–	100	GC
Zhang, 2023b (20)	China	Cross-sectional, P	WLI	240	33.8	–	18.3	GC
Yan, 2021 (21)	China	Case-control, P	WLI+NBI	324	28.9	–	100	GC
Ohno, 2020 (22)	Japan	Cross-sectional, R	WLI	162	32.1	–	100	GC
Kawamura, 2022 (23)	Japan	Cross-sectional, P	WLI+IEE	380	33.4	–	18.9	GC
Majima, 2019 (11)	Japan	Cross-sectional, R	WLI+LCI	194	27.3	–	100	GC
Matsumoto, 2024a (10)	Japan	Cross-sectional, R	WLI+IEE/NBI/LCI	328	40.2	64.1 (13.2)	100	GC/K-T
Matsumoto, 2024b (24)	Japan	Cross-sectional, P	WLI+NBI/BLI	93	50.5	57.9 (14.9)	100	GC/K-T
Moribata, 2016 (12)	Japan	Cohort, R	WLI	122	25.4	68.8 (8.6)	100	GC
Zhang, 2022 (8)	China	Cross-sectional, R	WLI	141	50.4	–	80.1	OLGA/OLGIM
Zhang, 2024 (9)	China	Cross-sectional, R	WLI+NBI	422	44.8	–	70.9	OLGA/OLGIM
Wang, 2023 (25)	China	Cross-sectional, R	WLI	182	56.4	56.5 (12.0)	–	OLGIM
Huang, 2020 (26)	China	Cross-sectional, R	WLI	166	33.1	–	100	K-T

R, retrospective; P, prospective; WLI, white light image; LCI, linked color imaging; NBI, narrow-band imaging; IEE, image-enhanced endoscopy; BLI, blue-laser imaging; GC, gastric cancer; K-T, Kimura–Takemoto classification; OLGA, Operative Link on Gastritis Assessment; OLGIM, Operative Link on Gastric Intestinal Metaplasia Assessment.

### 3.3 Methodological quality

All included studies were considered to have a low risk of bias. JBI scores for cross-sectional studies ranged from 6 to 8, while NOS scores for cohort and case-control studies ranged from 8 to 9. Detailed assessments were provided in **Supplementary Tables 3–S5**.

### 3.4 Association between MLR and GC

**Figure 2:**
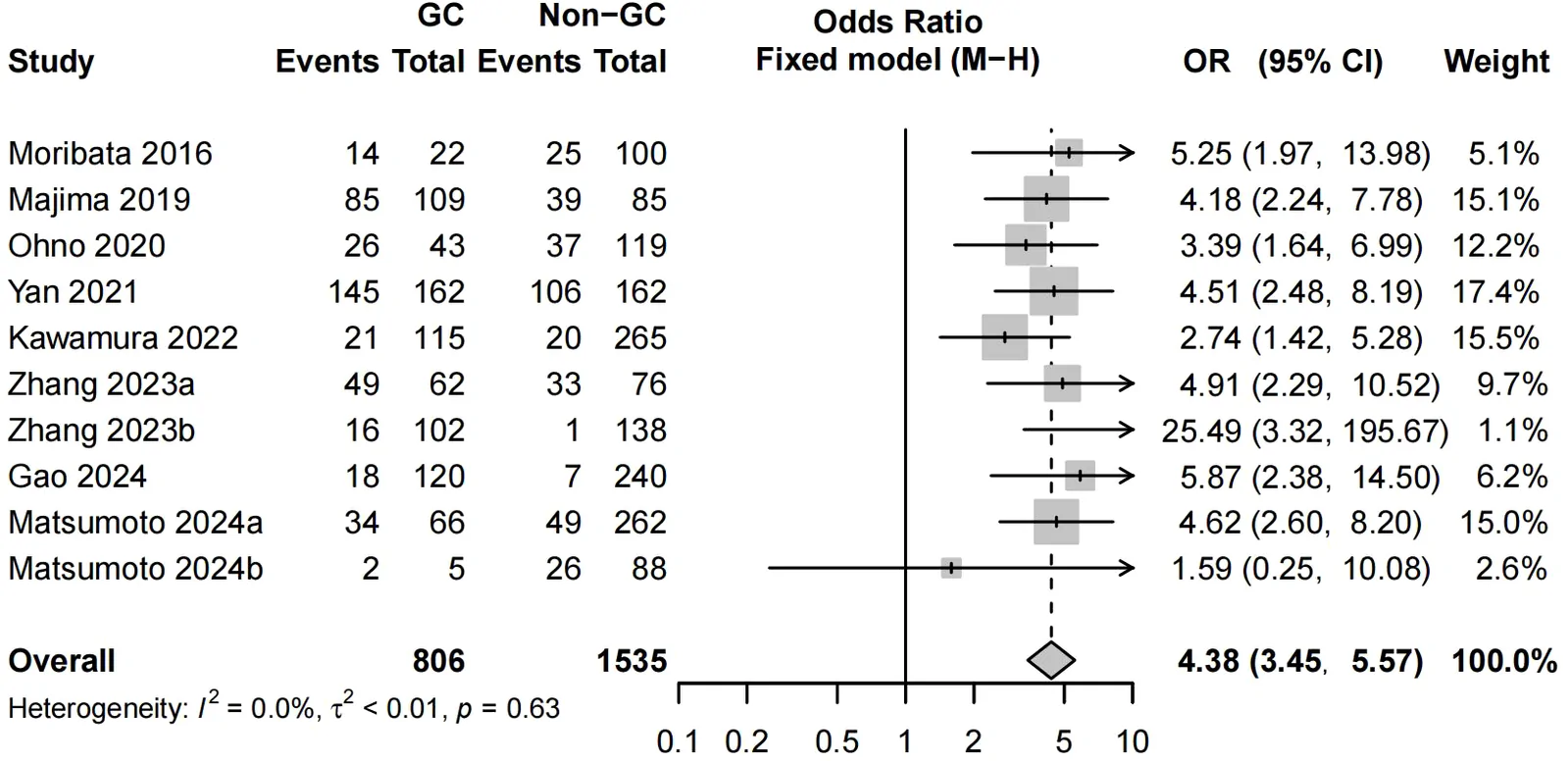
Forest plot of the association between map-like redness and gastric cancer.

Overall, we found that patients with MLR had a significantly higher risk of GC compared with subjects without MLR, with a pooled ORs of 4.38 (95% CI: 3.45–5.57) and negligible statistical heterogeneity (I^2^ = 0.0%) (**Figure 2**).

### 3.5 Association between MLR and high-risk precancerous lesions

**Figure 3:**
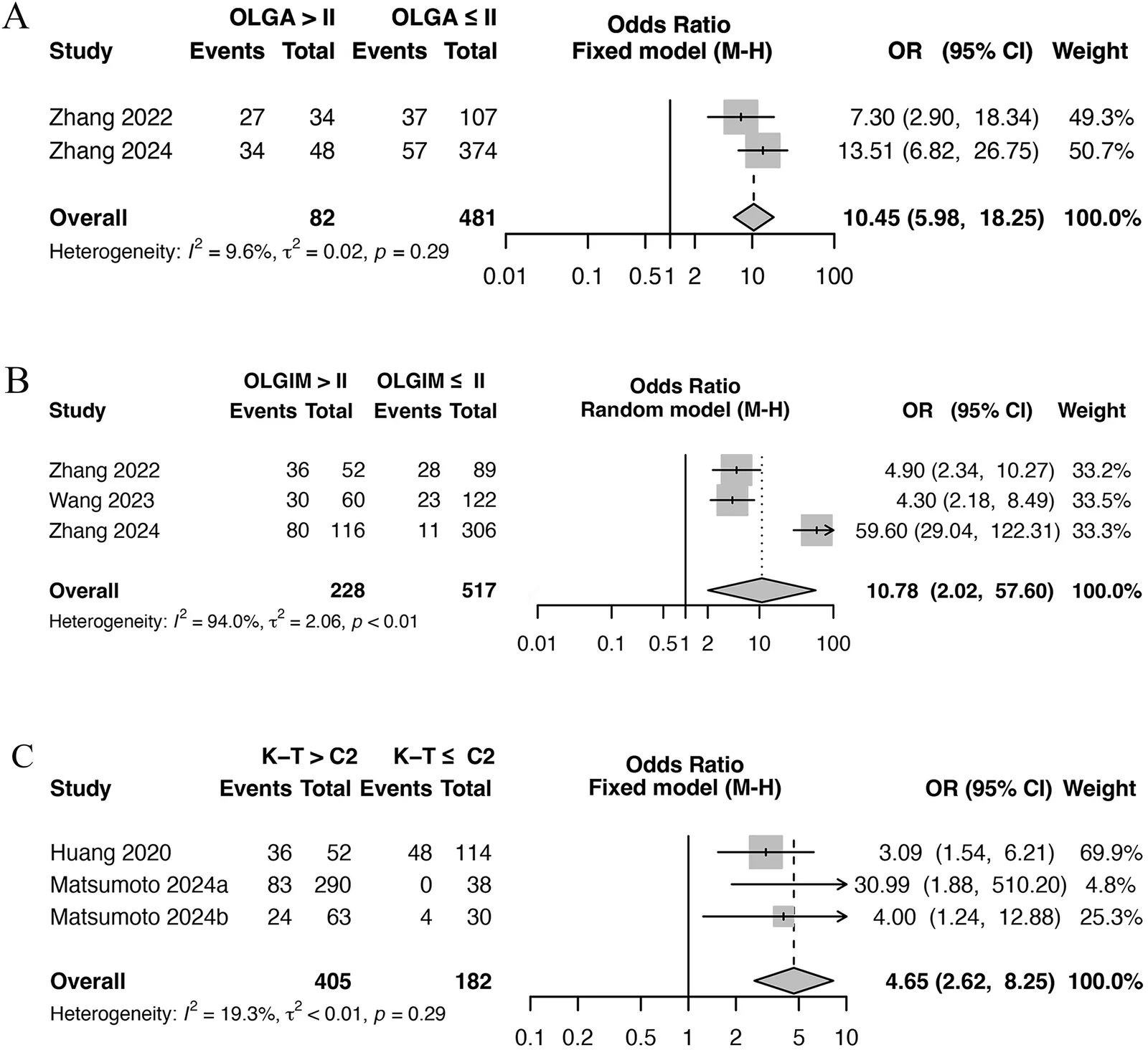
Forest plot of the association between map-like redness and high-risk precancerous lesions: **(A)** OLGA stage > II versus ≤ II; **(B)** OLGIM stage > II versus ≤ II; **(C)** Kimura–Takemoto classification > C2 versus ≤ C2.

MLR also shown strongly association with high-risk precancerous lesions: for OLGA stage > II, the pooled ORs was 10.45 (95% CI: 5.98–18.25), with low heterogeneity (I^2^ = 9.6%) (**Figure 3A**); for OLGIM stage > II, the pooled ORs was 10.78 (95% CI: 2.02–57.60), with substantial heterogeneity (I^2^ = 94.0%) (**Figure 3B**); and for K–T classification > C2, the pooled ORs was 4.65 (95% CI: 2.62–8.25), with low heterogeneity (I^2^ = 19.3%) (**Figure 3C**).

### 3.6 Validation of the meta-analysis

#### 3.6.1 Subgroup analysis

Predefined subgroup analyses demonstrated that the association between MLR and GC was strong and consistent, with no significant intergroup differences (**Table 2**). Geographically, the pooled ORs was 5.55 (95% CI: 3.70–8.31) for data from China, and 3.77 (95% CI: 2.80–5.08) for Japan. By study design, the pooled ORs was 5.25 (95% CI: 1.97–13.98) for cohort studies, 4.88 (95% CI: 3.21–7.41) for case-control studies, and 4.04 (95% CI: 2.98–5.49) for cross-sectional studies. In terms of time direction, the pooled ORs was 4.19 (95% CI: 2.79–6.29) for prospective and 4.50 (95% CI: 3.35–6.04) for retrospective designs. According to HP eradication status, the pooled ORs was 4.29 (95% CI: 3.26–5.64) for the complete eradication group and 5.31 (95% CI: 2.80–5.08) for the partial eradication group.

**Table 2:** Subgroup analysis: association between map-like redness and the risk of gastric cancer.

Subgroup	No. of studies	OR (95% CI)	I^2^ (%)	P subgroup
Geographic region				0.13
China	4	5.55 (3.70-8.31)	0	
Japan	6	3.77 (2.80-5.08)	0	
Study design				0.72
Cohort	1	5.25 (1.97-13.98)	–	
Case-control	3	4.88 (3.21-7.41)	0	
Cross-sectional	6	4.04 (2.98-5.49)	13.7	
Time direction				0.78
Prospective	4	4.19 (2.79-6.29)	45.2	
Retrospective	6	4.50 (3.35-6.04)	0	
H. pylori eradication^a^				0.68
Complete	7	4.29 (3.26-5.64)	0	
Partial	3	5.31 (2.80-5.08)	61.1	

^a^ Complete: all participants had prior H. pylori eradication; Partial: only a subset of participants had eradication.

#### 3.6.2 Sensitivity analysis

To assess the impact of any individual study on the overall result, a leave-one-out sensitivity analysis was conducted. The pooled ORs remained stable, ranging from 4.15 to 4.69 (**Supplementary Figure 1**), consistent with the main analysis and revealing high stability of our results. For the OLGIM > II outcome, an exploratory sensitivity analysis showed that, after excluding Zhang 2024 (9), heterogeneity decreased to 0% while the pooled association remained significant (ORs = 4.57, 95% CI 2.77–7.54; **Supplementary Figure 2**).

#### 3.6.3 Publication bias

Publication bias was assessed using a funnel plot. The plot showed a reasonably symmetric distribution, suggesting no substantial evidence of publication bias (**Supplementary Figure 3**). Egger’s weighted regression test also indicated no significant (P = 0.43).

## 4 Discussion

With increasing global awareness of the health burden posed by HP infection, HP eradication has become a key strategy for GC prevention. As a result, a growing number of individuals now fall into the post-eradication stage. However, HP eradication does not completely eliminate the risk of GC. On the one hand, it cannot reverse intestinal metaplasia or dysplasia that has already developed, and their natural progression may continue (27). On the other hand, existing studies have shown that the incidence of GC remains significantly higher in HP-eradicated individuals than in those who were never infected (28). Therefore, establishing effective risk stratification strategies for GC in the post-eradication population holds important clinical significance. In this context, MLR has received increasing attention as an easily recognizable and highly specific endoscopic feature in the post-eradication setting.

In general, the pooled results indicate that individuals with MLR have approximately a 4.4-fold increased risk of GC and a more than 10-fold increased risk of high-risk precancerous conditions (OLGA or OLGIM stage > II), pointing out a significant positive relationship between MLR and both GC and advanced precancerous lesions. These findings suggest that patients with MLR should be considered for targeted biopsies or shortened surveillance intervals, while the incorporation of MLR assessment into routine endoscopic evaluation could help clinicians identify high-risk individuals earlier and optimize strategies for GC risk stratification.

Although the pooled analysis of the primary outcome showed no statistical heterogeneity (I^2^ = 0%), predefined subgroup and sensitivity analyses were still conducted. In the subgroup analyses, the pooled ORs across different geographic regions (China and Japan), study designs (cohort, case-control, and cross-sectional), time direction (prospective vs. retrospective), and HP eradication status (complete vs. partial) ranged from 3.77 to 5.55, showing estimates comparable to the overall result with no significant differences observed between subgroups. In the sensitivity analysis, the pooled ORs after excluding each individual study ranged from 4.15 to 4.69, indicating no substantial impact on the overall conclusion. These findings collectively support the robustness of the present results and highlight the potential of MLR as a reliable risk marker for GC.

Several potential mechanisms may account for the association of MLR and GC. Histologically, MLR is considered a manifestation of extensive and severe intestinal metaplasia, which is often obscured by inflammation during HP infection and becomes endoscopically apparent after eradication, indicating its potential as a marker of high-risk mucosal areas (12). In terms of visual characteristics, GC arising after HP eradication often presents as depressed-type lesions (29), resembling the superficial concave appearance of MLR. Previous studies have reported anatomical overlap between MLR and the sites of newly developed or metachronous GC, suggesting that MLR may mark regions prone to malignant transformation (12, 13, 30). Moreover, given that MLR is most commonly observed along the lesser curvature of the corpus (10), which is one of the standard biopsy locations evaluated in the OLGIM staging system, its presence may elevate the overall OLGIM score and thereby influence the risk stratification of precancerous lesions. Regrettably, the mechanisms underlying the mucosal microenvironment of MLR remain poorly understood, particularly with respect to immune regulation, gene expression, and epigenetic alterations.

One other meta-analysis has previously evaluated the association between MLR and GC (31), reporting a pooled risk ratios of 2.34 (95% CI: 1.16–4.68; I^2^ = 96%), which favored the role of MLR in GC cases. However, several methodological limitations warrant attention. Most notably, one included study (Wei Y et al.) (32) used HP-positive GC patients as the control group, without including non-GC individuals, thereby making it impossible to determine whether MLR is associated with increased GC risk. This study also contributed substantially to the marked heterogeneity, as its effect direction was opposite to the other studies. Second, although substantial heterogeneity was observed, its sources were not further explored. Subgroup analysis was limited to the study design and did not account for other potential modifiers. In addition, there were errors in the study design classification. For example, a retrospective study by Moribata et al. (12) was misclassified as prospective, potentially undermining the validity of the subgroup analysis.

In contrast, our meta-analysis have several strengths. First, by incorporating eight additional studies (including 4 with GC outcomes) through updated English and Chinese database searches, we expanded the evidence base and showed greater benefit with better precision. Second, we not only assessed the association between MLR and GC but also included high-risk precancerous lesions as secondary outcomes, offering deeper insight into the role of MLR in stratifying GC risk after HP eradication. Finally, we obtained a homogeneous pooled estimate for the association between MLR and GC risk (I^2^ = 0). Despite the overall low heterogeneity, we performed prespecified subgroup and sensitivity analyses, which demonstrated consistent effect directions across different study settings, further supporting the robustness of our findings.

Our study did have several limitations. First, the majority of included studies adopted retrospective cross-sectional designs and lacked prospective, long-term follow-up data, thereby limiting causal inference between MLR and GC. Second, all included studies were conducted in China and Japan, resulting in a geographic concentration in East Asia and potential regional bias. This may limit the external validity of our findings, particularly in Western populations where HP epidemiology and GC phenotypes differ substantially from those in East Asia (33, 34). Further multicenter, cross-regional, and prospective longitudinal studies are still needed to validate these findings and strengthen causal inference. Third, although MLR is readily identifiable under endoscopy as a post-HP eradication feature, no standardized evaluation criteria have been established. Most studies classified MLR simply as present or absent, without detailed stratification based on its quantity, extent, or anatomical location. For transparency, MLR definitions in the included studies are summarized in **Supplementary Table 6**. At last, proton pump inhibitors (PPIs) are associated with both MLR and GC (10, 35). However, relevant information on PPIs use, such as frequency, dosage, and duration of discontinuation, is generally lacking, which limits the feasibility of subgroup analyses.

In conclusion, our meta-analysis indicates that individuals with MLR are significantly associated with an increased risk of developing GC and high-risk precancerous lesions. This association remained consistent and strong across subgroup analyses stratified by geographic region, study design, and HP eradication status. Therefore, we recommend that large-scale, long-term, and well-designed prospective studies should be conducted to establish evidence-based endoscopic surveillance strategies for MLR and to further incorporate MLR as a positive endoscopic marker into GC risk stratification frameworks following HP eradication in the future.

## Data Availability

The raw data supporting the conclusions of this article will be made available by the authors, without undue reservation.
