# Correction: Association between map-like redness and the risk of gastric cancer and high-risk precancerous lesions: a systematic review and meta-analysis

**DOI:** 10.3389/fonc.2026.1987835

**Published:** 2026-10-19

**Authors:** Baixiang He, Mingyue Liu, Xiaochi Ma, Sheng Huang, Xiangyun Zou, Liju Zhang, Xiyan Zhang, Tong Li, Jinglin Zhang, Zhihong Li

**Affiliations:** 1 Gastroenterology Department, Dongzhimen Hospital, Beijing University of Chinese Medicine, Beijing, China; 2 Suzhou Hospital, Xiyuan Hospital of China Academy of Chinese Medical Sciences, Suzhou, China; 3 Dermatology Department, Xiyuan Hospital, Beijing, China; 4 Gastroenterology Department, The First Affiliated Hospital, Henan University of Chinese Medicine, Zhengzhou, China

**Keywords:** endoscopic marker, gastric cancer, map-like redness, meta-analysis, post-*Helicobacter pylori* eradication

The affiliation "Suzhou Hospital, Xiyuan Hospital of China Academy of Chinese Medical Sciences, Suzhou, China" was omitted from the paper. This affiliation has now been added for author Mingyue Liu and is listed as her first affiliation. Her previously published affiliation, "Dermatology Department, Xiyuan Hospital, Beijing, China," has been retained as an additional affiliation. Accordingly, the affiliation numbering has been updated: Mingyue Liu is affiliated with affiliations 2 and 3, and Xiaochi Ma with affiliation 4; all other author-affiliation mappings remain unchanged.

The original version of this article has been updated.

