# Supplementary material for: Association between map-like redness and the risk of gastric cancer and high-risk precancerous lesions: a systematic review and meta-analysis
Source: Front Oncol. 2026 Apr 22;16:1760001. doi: 10.3389/fonc.2026.1760001 (PMC13143535; doi:10.3389/fonc.2026.1760001)
Supplement: Supplementary file 1 [file DataSheet1.docx]

**Association between map-like redness and the risk of gastric cancer and high-risk precancerous lesions: a systematic review and meta-analysis**

Bai-xiang He*, Ming-yue Liu*, Xiao-chi Ma*, Sheng Huang, Xiang-yun Zou, Li-ju Zhang, Xi-yan Zhang, Tong Li, Jing-lin Zhang, Zhi-hong Li

**Contents**

**Table S1 MOOSE checklist Page 2-3**

**Table S2 Search strategies Page 4-5**

**Table S3 Results of quality assessment of cross-sectional studies Page 5**

**Table S4 Results of quality assessment of case–control studies Page 5**

**Table S5 Results of quality assessment of cohort studies Page 6**

**Table S6 Summary of the Diagnostic Criteria for Map-Like Redness Page 6**

**Figure S1 Sensitivity analysis after each study was excluded Page** **7**

**Figure S2** **Sensitivity analysis for the OLGIM > II outcome Page 7**

**Figure S2 Funnel plots Page 7**

**Table S1 MOOSE Checklist for Meta-analyses of Observational Studies**

| **Item No** | **Recommendation** | **Reported on Page No** |
| --- | --- | --- |
| Reporting of background should include | | |
| 1 | Problem definition | 3 |
| 2 | Hypothesis statement | 4 |
| 3 | Description of study outcome(s) | 4 |
| 4 | Type of exposure or intervention used | 6 |
| 5 | Type of study designs used | 6 |
| 6 | Study population | 6 |
| Reporting of search strategy should include | | |
| 7 | Qualifications of searchers (eg, librarians and investigators) | 5 |
| 8 | Search strategy, including time period included in the synthesis and key words | 5 |
| 9 | Effort to include all available studies, including contact with authors | 5 |
| 10 | Databases and registries searched | 5 |
| 11 | Search software used, name and version, including special features used (eg, explosion) | 5 |
| 12 | Use of hand searching (eg, reference lists of obtained articles) | 5 |
| 13 | List of citations located and those excluded, including justification | Fig.1 |
| 14 | Method of addressing articles published in languages other than English | 5 |
| 15 | Method of handling abstracts and unpublished studies | - |
| 16 | Description of any contact with authors | 6 |
| Reporting of methods should include | | |
| 17 | Description of relevance or appropriateness of studies assembled for assessing the hypothesis to be tested | 6 |
| 18 | Rationale for the selection and coding of data (eg, sound clinical principles or convenience) | 7 |
| 19 | Documentation of how data were classified and coded (eg, multiple raters, blinding and interrater reliability) | 7 |
| 20 | Assessment of confounding (eg, comparability of cases and controls in studies where appropriate) | 7 |
| 21 | Assessment of study quality, including blinding of quality assessors, stratification or regression on possible predictors of study results | 7 |
| 22 | Assessment of heterogeneity | 8 |
| 23 | Description of statistical methods (eg, complete description of fixed or random effects models, justification of whether the chosen models account for predictors of study results, dose-response models, or cumulative meta-analysis) in sufficient detail to be replicated | 8 |
| 24 | Provision of appropriate tables and graphics | Table S2 |
| Reporting of results should include | | |
| 25 | Graphic summarizing individual study estimates and overall estimate | 9 |
| 26 | Table giving descriptive information for each study included | Table 1 |
| 27 | Results of sensitivity testing (eg, subgroup analysis) | 10, Fig. S1 |
| 28 | Indication of statistical uncertainty of findings | 9 |

| **Item No** | **Recommendation** | **Reported on Page No** |
| --- | --- | --- |
| Reporting of discussion should include | | |
| 29 | Quantitative assessment of bias (eg, publication bias) | 11 |
| 30 | Justification for exclusion (eg, exclusion of non-English language citations) | Fig.1 |
| 31 | Assessment of quality of included studies | 9, Table S3-S5 |
| Reporting of conclusions should include | | |
| 32 | Consideration of alternative explanations for observed results | 13-14 |
| 33 | Generalization of the conclusions (ie, appropriate for the data presented and within the domain of the literature review) | 14 |
| 34 | Guidelines for future research | 15 |
| 35 | Disclosure of funding source | 1 |

**Table S2. Searching strategies**

**a. for Cochrane Library**

| **Search number** | **Query** |
| --- | --- |
| **1** | (map-like redness):ab,ti,kw OR (patchy redness):ab,ti,kw OR (Kyoto classification of gastritis):ab,ti,kw OR (Kyoto classification):ab,ti,kw |
| **2** | MeSH descriptor: [Stomach Neoplasms] explode all trees |
| **3** | (Stomach Neoplasms):ti,ab,kw OR (Gastric Cancer):ab,ti,kw OR (Cancer of the Stomach):ab,ti,kw OR (Cancer, Gastric):ab,ti,kw OR (Cancer, Stomach):ab,ti,kw OR (Stomach Cancer):ab,ti,kw OR (Cancer of Stomach):ab,ti,kw OR (Neoplasm, Gastric):ab,ti,kw OR (Gastric Neoplasm):ab,ti,kw OR (Neoplasm, Stomach):ab,ti,kwOR (Stomach Neoplasm):ab,ti,kw |
| **4** | (early gastric cancer):ab,ti,kw OR (EGC):ab,ti,kw OR (gastric adenocarcinoma):ab,ti,kw OR (adenocarcinoma of the stomach):ab,ti,kw OR (OLGA):ab,ti,kw OR (OLGIM):ab,ti,kw OR (operative link on gastritis assessment):ab,ti,kw OR (operative link on gastritis intestinal metaplasia assessment):ab,ti,kw |
| **5** | #2 OR #3 OR #4 |
| **6** | #1 AND #5 |

**b. for Embase**

| **Search number** | **Query** |
| --- | --- |
| **1** | 'map-like redness':ti,ab,kw OR 'patchy redness':ti,ab,kw OR 'kyoto classification of gastritis':ti,ab,kw OR 'kyoto classification':ti,ab,kw |
| **2** | 'stomach tumor'/exp |
| **3** | 'gastric mass (tumor)':ti,ab,kw OR 'gastric masses (tumor)':ti,ab,kw OR 'gastric neoplasia':ti,ab,kw OR 'gastric neoplasm':ti,ab,kw OR 'gastric subepithelial tumor':ti,ab,kw OR 'gastric tumor':ti,ab,kw OR 'gastric tumorigenesis':ti,ab,kw OR 'gastric tumour':ti,ab,kw OR 'mucosa tumor, stomach':ti,ab,kw OR 'mucosa tumour, stomach':ti,ab,kw OR 'neoplasia of the stomach':ti,ab,kw OR 'neoplasm of the stomach':ti,ab,kw OR 'neoplasms of the stomach':ti,ab,kw OR 'neoplastic gastric':ti,ab,kw OR 'neoplastic stomach':ti,ab,kw OR 'stomach mucosa tumor':ti,ab,kw OR 'stomach mucosa tumour':ti,ab,kw OR 'stomach neoplasia':ti,ab,kw OR 'stomach neoplasm':ti,ab,kw OR 'stomach tumour':ti,ab,kw OR 'stomach ulcerated tumor':ti,ab,kw OR 'stomach ulcerated tumour':ti,ab,kw OR 'stomach ulcerating tumor':ti,ab,kw OR 'stomach ulcerating tumour':ti,ab,kw OR 'tumor of the gastric':ti,ab,kw OR 'tumor of the stomach':ti,ab,kw OR 'tumor, stomach mucosa':ti,ab,kw OR 'tumour of the gastric':ti,ab,kw OR 'tumour of the stomach':ti,ab,kw OR 'tumour, stomach mucosa':ti,ab,kw OR 'stomach tumor':ti,ab,kw |
| **4** | 'early gastric cancer':ti,ab,kw OR 'egc':ti,ab,kw OR 'gastric adenocarcinoma':ti,ab,kw OR 'adenocarcinoma of the stomach':ti,ab,kw OR 'olga':ti,ab,kw OR 'olgim':ti,ab,kw OR 'operative link on gastritis assessment':ti,ab,kw OR 'operative link on gastritis intestinal metaplasia assessment':ti,ab,kw |
| **5** | #2 OR #3 OR #4 |
| **6** | #1 AND #5 |

**Note: For the Chinese search strategies, please contact the authors if needed.**

**Table S3** Results of quality assessment of cross-sectional studies (JBI).

| Study ID | Q.1 | Q.2 | Q.3 | Q.4 | Q.5 | Q.6 | Q.7 | Q.8 | Total | ROB |
| --- | --- | --- | --- | --- | --- | --- | --- | --- | --- | --- |
| Zhang 2024 | 1 | 1 | 1 | 1 | 1 | 0 | 1 | 1 | 7 | Low |
| Matsumoto 2024a | 1 | 1 | 1 | 1 | 1 | 0 | 1 | 1 | 7 | Low |
| Matsumoto 2024b | 1 | 1 | 1 | 1 | 1 | 0 | 1 | 1 | 7 | Low |
| Zhang 2023b | 1 | 1 | 1 | 1 | 1 | 1 | 1 | 1 | 8 | Low |
| Wang 2023 | 1 | 1 | 1 | 1 | 0 | 0 | 1 | 1 | 6 | Low |
| Zhang 2022 | 1 | 1 | 1 | 1 | 1 | 0 | 1 | 1 | 7 | Low |
| Masashi 2022 | 1 | 1 | 1 | 1 | 1 | 0 | 1 | 1 | 7 | Low |
| Huang 2020 | 1 | 1 | 1 | 1 | 1 | 0 | 1 | 1 | 7 | Low |
| Ohno 2020 | 1 | 1 | 1 | 1 | 1 | 0 | 1 | 1 | 7 | Low |
| Majima 2019 | 1 | 1 | 1 | 1 | 1 | 1 | 1 | 1 | 8 | Low |

Q.1: Were the criteria for inclusion in the sample clearly defined? Q.2: Were the study subjects and the setting described in detail? Q.3: Was the exposure measured in a valid and reliable way? Q.4: Were objective, standard criteria used for measurement of the condition? Q.5: Were confounding factors identified? Q.6: Were strategies to deal with confounding factors stated? Q.7: Were the outcomes measured in a valid and reliable way? Q.8: Was appropriate statistical analysis used? ROB, Risk of Bias.

The number 0 means that the item is no or unclear.

**Table S4** Results of quality assessment of case–control studies (NOS for case–control).

| Study ID | Q.1 | Q.2 | Q.3 | Q.4 | Q.5 | Q.6 | Q.7 | Q.8^a^ | Total | ROB |
| --- | --- | --- | --- | --- | --- | --- | --- | --- | --- | --- |
| Gao 2024 | 1 | 1 | 1 | 1 | 1 | 1 | 1 | 1 | 8 | Low |
| Zhang 2023a | 1 | 1 | 1 | 1 | 2 | 1 | 1 | 1 | 9 | Low |
| Yan 2021 | 1 | 1 | 1 | 1 | 2 | 1 | 1 | 1 | 9 | Low |

Q.1: Is the Case Definition Adequate? Q.2: Representativeness of the Cases. Q.3: Selection of Controls. Q.4: Definition of Controls. Q.5: Comparability of cases and controls on the basis of the design or analysis. Q.6: Ascertainment of exposure. Q.7: Same method of ascertainment for cases and controls. Q.8: Non-Response rate.

^a^Lost to follow-up rate considered acceptable if <5%.

**Table S5** Results of quality assessment of cohort studies (NOS for cohort)**.**

| Study ID | Q.1 | Q.2 | Q.3 | Q.4 | Q.5 | Q.6 | Q.7^b^ | Q.8 | Total | ROB |
| --- | --- | --- | --- | --- | --- | --- | --- | --- | --- | --- |
| Moribata 2016 | 1 | 1 | 1 | 1 | 2 | 1 | 1 | 1 | 9 | Low |

Q.1: Representativeness of the exposed cohort. Q.2: Selection of the non exposed cohort Q.3: Ascertainment of exposure. Q.4: Demonstration that outcome of interest was not present at start of study. Q.5: Comparability of cohorts on the basis of the design or analysis. Q.6: Assessment of outcome. Q.7: Was follow-up long enough for outcomes to occur. Q.8: Adequacy of follow up of cohorts.

^b^Follow-up time considered adequate if ≥3 years.

**Table S6 Summary of the Diagnostic Criteria for Map-Like Redness**

| Study ID | Diagnostic Criteria |
| --- | --- |
| Huang 2020 | Kyoto Classification of Gastritis |
| Wang 2023 | Kyoto Classification of Gastritis |
| Zhang 2022 | Kyoto Classification of Gastritis |
| Zhang 2024 | Kyoto Classification of Gastritis |
| Gao 2024 | Kyoto Classification of Gastritis |
| Kawamura 2022 | Not reported |
| Majima 2019 | An erythematous lesion, shallowly depressed and clearly demarcated from the background mucosa, with variable size and color tone |
| Matsumoto 2024-a | A shallowly depressed erythematous lesion distinct from the background mucosa |
| Matsumoto 2024-b | A shallowly depressed erythematous lesion distinct from the background mucosa |
| Moribata 2016 | Flat or depressed erythematous geographical lesions. |
| Ohno 2020 | Not reported |
| Yan 2021 | Kyoto Classification of Gastritis |
| Zhang 2023-a | A flat or slightly depressed lesion with variable morphology, size, and erythema, which may merge into a large map-like area or present as small, discontinuous patchy lesions. |
| Zhang 2023-b | Kyoto Classification of Gastritis |


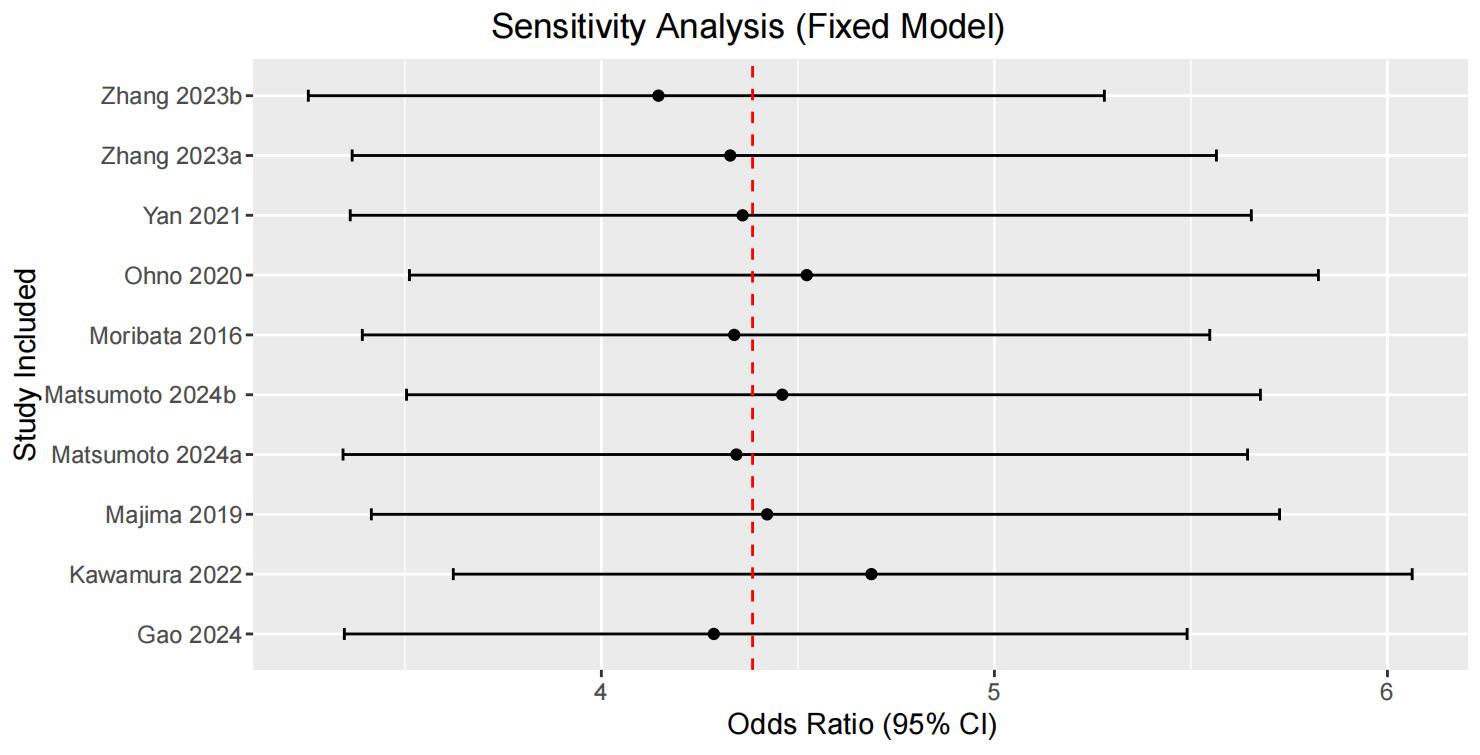


**Figure S1** Sensitivity analysis after each study was excluded.


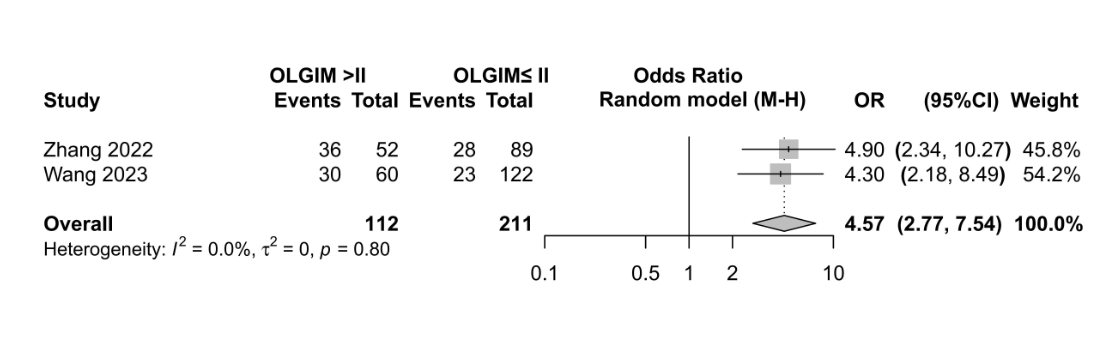


**Figure S2** Sensitivity analysis for the OLGIM > II outcome


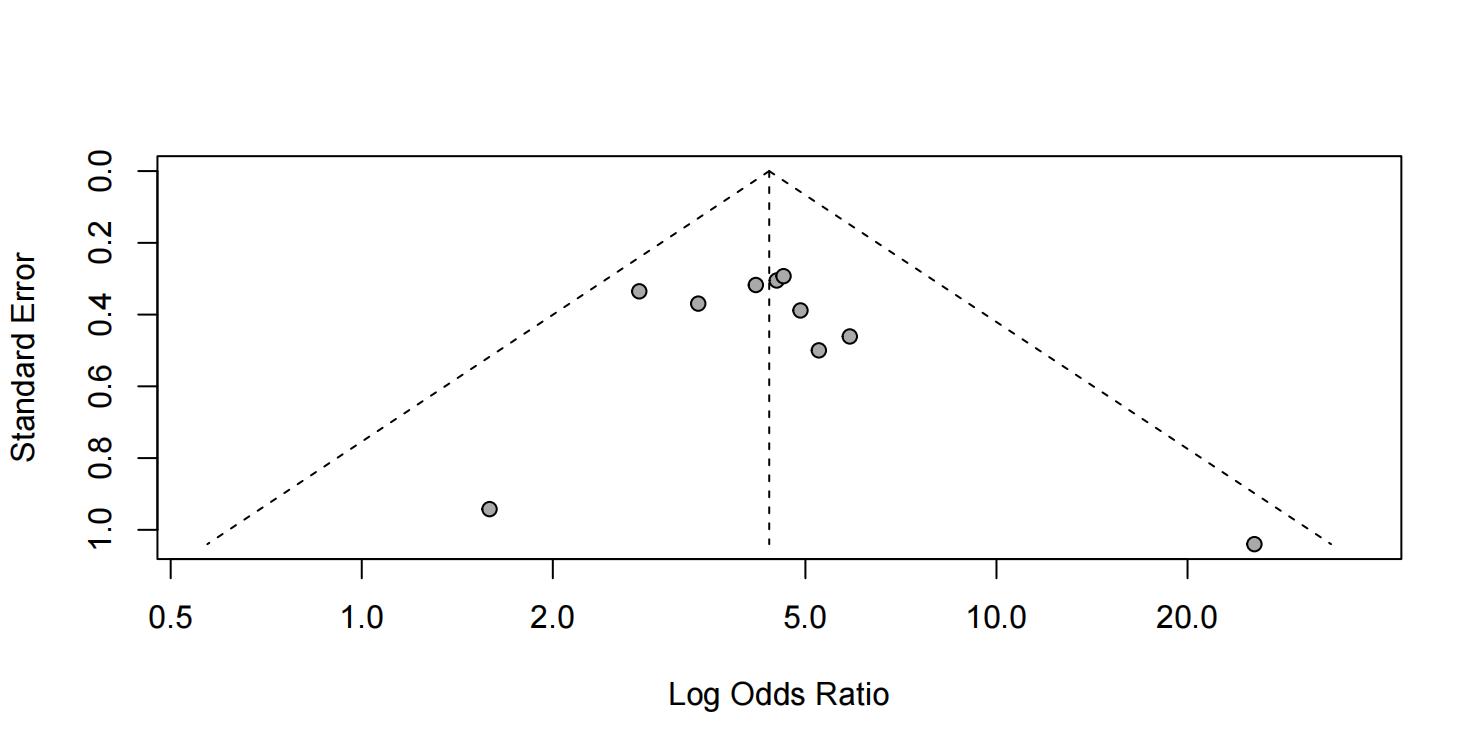


**Figure S3** Funnel plots.
